# The relationship between social capital, self-efficacy, and health-related quality of life in rural empty-nest older adults with chronic diseases: a latent profile and mediation analysis

**DOI:** 10.3389/fpubh.2026.1866381

**Published:** 2026-08-21

**Authors:** Yajuan Wang, Yu Zhao, Chunyu Tian, Limin Shi, Mengze Xu

**Affiliations:** 1 Department of Nursing, Chengde Medical University, Chengde, Hebei, China; 2 Department of Breast Surgery, Affiliated Hospital of Chengde Medical University, Chengde, Hebei, China

**Keywords:** health-related quality of life, latent profile analysis, mediation analysis, rural empty-nest older adults, self-efficacy, social capital

## Abstract

**Background:**

Health-related quality of life is a key indicator of health status among rural empty-nest older adults with chronic diseases, who face limited social resources and a high disease burden. Although social capital is associated with quality of life, its heterogeneity and underlying mechanisms remain unclear. This study aims to identify distinct profiles of social capital using latent profile analysis, examine their effects on health-related quality of life, and test the mediating role of self-efficacy.

**Methods:**

A total of 425 rural empty-nest older adults with chronic diseases in Ningxia, China were surveyed between April and December 2025 using multistage stratified random sampling method combined with convenience sampling. Data were collected using the social capital scale, the self-efficacy scale, and the EuroQol five-dimension questionnaire (EQ-5D). All statistical analyses were performed using SPSS 27.0 and Mplus 8.3.

**Results:**

Latent profile analysis identified three social capital profiles: low (29.9%), moderate (49.4%), and high (20.7%). Health-related quality of life and self-efficacy differed significantly across profiles (*P* < 0.001). In mediation analyses using the low profile as the reference, the indirect effect of self-efficacy was not supported for the moderate profile (SE =0.014, 95% CI =-0.002, 0.053). In contrast, a significant indirect effect was observed for the high profile (SE =0.017, 95% CI =0.028, 0.095), with self-efficacy accounting for 21.2% of the total effect.

**Conclusion:**

Social capital was heterogeneous among rural empty-nest older adults with chronic diseases. The high social capital profile was associated with higher health-related quality of life and self-efficacy than the lower profiles. Self-efficacy partially mediated this association, whereas no significant indirect effect was found for the moderate profile. These findings support profile-informed interventions that enhance self-efficacy in individuals with high social capital and strengthen social capital in those with low or moderate social capital.

## 1 Introduction

China is experiencing rapid population aging, accompanied by a growing proportion of older adults living as “empty nesters.” Empty-nest older adults are defined as individuals aged 60 years or older who have no children or whose children have been absent for a prolonged period, and who live alone or only with their spouse (1). Based on data from the Seventh National Population Census and related demographic projections, the prevalence of empty-nesting is expected to increase substantially by 2030, potentially reaching approximately 90%, which would correspond to more than 200 million older adults. This phenomenon is particularly common in rural areas, where economic resources are relatively limited and formal support systems remain underdeveloped (2). At the same time, the health challenges of older adults are increasingly dominated by the high burden of chronic diseases and multimorbidity. Global projections suggest that the proportion of people aged ≥ 65 years will rise to 16% by 2050, and that approximately 85% of older adults will have at least one chronic condition, with around 60% living with two or more (3). In China, the prevalence of chronic diseases among adults aged ≥60 years has been reported to reach 86.39%, and 66.30% have multimorbidity (4). Chronic diseases contribute to approximately 70% of disability-adjusted life years lost and 80% of deaths among older adults (5). The Healthy China 2030 initiative underscores the importance of promoting healthy aging, with the overarching aim of improving quality of life and extending healthy life expectancy among older adults (6). Within this context, the health status of rural empty-nest older adults with chronic diseases constitutes a critical gap that must be addressed to achieve these policy goals.

Health-related quality of life (HRQoL) reflects individuals' subjective perceptions of their well-being in relation to their health status and living circumstances. As a multidimensional construct, HRQoL encompasses physical, psychological, and social functioning and serves as an overall indicator of health and well-being (7). In rural Ningxia in western China, an economically underdeveloped region, empty-nest older adults with chronic diseases often experience a dual burden of physical illness and psychological distress, and their HRQoL remains poor (8). At present, the HRQoL of this group has not received sufficient attention. Most studies have focused on the general older population (9) or on older adults with chronic diseases in urban settings (10), and existing interventions are often broad and insufficiently tailored to the needs of rural empty-nest older adults (11). Accordingly, further research is warranted to identify key determinants of HRQoL in this group and to clarify the mechanisms through which these factors influence health outcomes.

Triadic reciprocal determinism, a core component of social cognitive theory, provides an integrative analytical framework to address the above issues (12). The theory posits that individual factors, environmental factors, and behavior influence each other and are reciprocally causal. In the context of the present study, environmental factors correspond to an individual's social capital, including social networks, trust, and reciprocity; individual factors correspond to self-efficacy, defined as an individual's confidence in performing health management behaviors; and health-related quality of life is conceptualized as a composite outcome of behavior and functional status (13). Based on this theory, external environmental resources (social capital) may not only directly affect health outcomes but also indirectly improve HRQoL by influencing an individual's self-efficacy.

Specifically, self-efficacy refers to an individual's confidence in their ability to carry out health-management behaviors. As a key psychological determinant (12), it has been consistently associated with better HRQoL (7). Among rural empty-nest older adults with chronic diseases, self-efficacy may be particularly important, helping to mitigate psychological distress and promote engagement in health-promoting behaviors (14). From the perspective of triadic reciprocal determinism, self-efficacy (an individual factor) serves not only as a mediator through which environmental factors influence behavioral outcomes but also reciprocally affects an individual's perception and utilization of environmental resources.

Social capital, an important external resource for coping with chronic illness, emphasizes the role of social networks, trust, and reciprocity in facilitating access to support and resources (15). Through social support, social participation, interpersonal trust, and access to material resources, social capital may influence health both directly and indirectly (16). A growing body of evidence links social capital to higher HRQoL (17, 18). Within the framework of triadic reciprocal determinism, social capital (environmental factor) may influence HRQoL (behavioral outcome) through two pathways: a direct positive effect, and an indirect pathway mediated by self-efficacy (individual factor). However, most existing studies have adopted variable-centered approaches that overemphasize the overall effect of social capital, rarely explore its internal heterogeneity, and neglect individual differences across different patient subgroups. As a result, the complex mechanisms through which social capital affects HRQoL remain poorly understood.

Latent profile analysis (LPA), a person-centered method (19), can identify distinct patterns of social capital subgroups among rural empty-nest older adults with chronic diseases. In the context of triadic reciprocal determinism, different latent profiles of social capital can be interpreted as manifestations of varying “environment-individual” interaction patterns (17). Thus, this approach becomes key to deeply understanding the heterogeneity in HRQoL and the underlying mechanisms across older adults with different types of social capital, while also informing the development of targeted interventions. However, evidence on the interrelationships among HRQoL, self-efficacy, and social capital remains limited, particularly among rural empty-nest older adults with chronic diseases.

Based on the above theoretical framework and empirical evidence, this study focuses on rural empty-nest older adults with chronic diseases, aiming to identify the heterogeneity of social capital in this population, examine the effect of different latent profiles of social capital on HRQoL, and test the mediating role of self-efficacy between social capital latent profiles and HRQoL. Accordingly, we proposed the following hypotheses: (1) social capital in this population can be characterized by distinct latent profiles; (2) HRQoL differs across these profiles; (3) self-efficacy mediates the association between social-capital profiles and HRQoL.

## 2 Methods

### 2.1 Design and participants

A combined multi-stage stratified random sampling and convenience sampling approach was employed (20). In the first stage, based on the classification of economic development levels in Ningxia (high, medium, and low) (11), one prefecture-level city was randomly selected from each stratum: Yinchuan, Wuzhong, and Guyuan. In the second stage, from April to December 2025, two counties were randomly selected within each city, and one administrative village was then identified in each county. Eligible rural empty-nest older adults were subsequently recruited using convenience sampling, yielding a final sample of 425 participants. This study was conducted in accordance with the Declaration of Helsinki and was approved by the Ethics Committee of Chengde Medical University (No. 2025015).

Inclusion criteria were: (1) age ≥ 60 years; (2) living alone or with a spouse, with no children or with children not living nearby; (3) residence in rural areas for ≥1 year; (4) Individuals diagnosed with chronic diseases in hospitals at or above the county (district) level; and (5) provision of informed consent. Exclusion criteria were: (1) Individuals with visual or hearing impairments that hindered participation; (2) Those unable to complete the survey due to mental illness or other conditions.

### 2.2 Sample size

Although no standardized a priori power calculation exists for latent profile analysis (LPA), the sample size was justified based on established methodological recommendations. Previous simulation studies suggest that at least 300 participants are generally sufficient to obtain stable LPA solutions and accurate profile classification (21). After allowing for a 20% rate of invalid or incomplete questionnaires, the minimum sample size required for LPA was 375. For the mediation analysis, Kendall's rule of thumb recommends that the required sample size should be 5–10 times the number of variables included in the model (22). Given the 22 variables included in this study, the required sample size ranged from 110 to 220. Therefore, the target sample size was set at no fewer than 375 participants. Ultimately, 425 eligible participants were included in the final analysis.

### 2.3 Instrument

#### 2.3.1 Demographic characteristics

The questionnaire was self-developed by the researchers based on a literature review and discussions within the research team. It included demographic information (sex, age, ethnicity, educational level, marital status, family monthly income per capita, medical insurance and previous occupation), body mass index (BMI), exercise frequency, smoking and alcohol consumption, and disease-related information (self-rated sleep quality, number of chronic diseases, and disease duration). In accordance with the inclusion criteria, empty-nest status was defined as living alone or living with a spouse only. Among the participants, 218 lived alone and 207 lived with a spouse only.

Self-rated sleep quality was assessed using a brief subjective evaluation developed by the research team with reference to the National Sleep Foundation's (23) sleep time duration recommendations and the Chinese expert consensus (24) on sleep health and rehabilitation management for community-dwelling older adults. Sleep quality was classified into three categories: poor, fair, and good. Poor: sleep duration < 5 h, sleep latency >60 min, nocturnal awakenings ≥4 times, and obvious daytime fatigue; Fair: sleep duration of 5–6 h, sleep latency of 31–60 min, nocturnal awakenings 3 times, and mild daytime fatigue after waking; Good: sleep duration of 7–8 h, sleep latency ≤ 30 min, nocturnal awakenings 0–2 times, and relatively good daytime energy after waking.

#### 2.3.2 Social capital scale

The 2018 version of the social capital scale developed by Xu Xiaoru was used (25). The scale comprises 22 items across six dimensions: social participation, social connection, social support, reciprocity, trust, and sense of belonging. All items are rated on a 5-point Likert scale, yielding a total score ranging from 22 to 110; higher total scores indicate better social capital. In the present study, Cronbach's α for the scale was 0.907.

#### 2.3.3 EuroQol five-dimension questionnaire (EQ-5D)

The EQ-5D was adopted, which consists of five dimensions: mobility, self-care, daily activities, pain/discomfort, and anxiety/depression. Each dimension has five levels. In this study, the EQ-5D health utility value scoring system established by Luo et al. (26) based on the Chinese population was used. The utility score ranges from −0.391 to 1, with higher values indicating better quality of life. In this study, Cronbach's α for the scale was 0.937.

#### 2.3.4 General self-efficacy scale (GSES)

Self-efficacy was assessed using the Chinese version of the General Self Efficacy Scale (GSES), translated and culturally adapted by Wang Caikang et al. (27). The GSES is a 10-item, single-factor instrument. Items are rated on a 4-point Likert scale from 1 (not at all true) to 4 (exactly true), producing a total score ranging from 10 to 40, with higher scores indicating greater self-efficacy. In this study, Cronbach's α for the scale was 0.939.

### 2.4 Data collection

After undergoing unified training and obtaining the informed consent from the participants, the investigators conducted one-on-one surveys using electronic questionnaires designed and distributed through the Wenjuanxing online survey platform. During the survey process, investigators provided on-site explanations when necessary and checked the completeness of the questionnaires in real time. The electronic questionnaire was completed on mobile devices, and the survey data were collected and exported through the Wenjuanxing system. After the data were exported, two researchers checked the data and eliminated the invalid questionnaires. A total of 425 valid questionnaires were collected, with an effective recovery rate of 92.21%.

### 2.5 Data analysis

Data were analyzed using SPSS 27.0 (IBM Corp., Armonk, NY, USA) and Mplus 8.3 (Muthén & Muthén, Los Angeles, CA, USA). Descriptive statistics were performed in SPSS 27.0, with categorical variables presented as frequencies and percentages (%) and continuous variables described as the median and interquartile range [M (P25, P75)]. To evaluate variations in health utility scores based on participant demographics, the Mann–Whitney *U* test was employed for comparing two distinct groups, while the Kruskal–Wallis *H* test was utilized for analyses involving three or more groups. Spearman's rank correlation analysis was then applied to examine the interrelationships between social capital, health-related quality of life, and self-efficacy. Subsequently, LPA was conducted in Mplus 8.3 to identify latent subgroups with distinct social-capital characteristics; models with increasing numbers of profiles were fitted and evaluated using the Akaike information criterion (AIC), Bayesian information criterion (BIC), sample-size adjusted BIC (aBIC), entropy, the Lo–Mendell–Rubin likelihood ratio test (LMR-LRT), the bootstrap likelihood ratio test (BLRT), and posterior class membership probabilities. Lower AIC, BIC, and aBIC values signify improved model fit, while entropy values approaching 1 reflect greater classification accuracy; an entropy value exceeding 0.80 was regarded as indicating satisfactory classification (28). Univariate and multivariable regression analyses were conducted to explore factors associated with the latent profile analysis-derived subgroups. The Kruskal–Wallis *H* test was employed to compare levels of self-efficacy and HRQoL among the identified profiles. Finally, mediation analysis was conducted using Hayes' PROCESS macro (Model 4) in SPSS 27.0 to test whether self-efficacy mediated the association between social-capital profiles and health-related quality of life; sociodemographic and disease-related variables associated with health-related quality of life, as well as variables associated with profile membership (*P* < 0.05), were included as covariates in the final models. Statistical significance was set at a two-tailed *P*-value < 0.05.

## 3 Results

### 3.1 Demographic characteristics

A total of 425 rural empty-nest older adults with chronic diseases were included in this study. The mean age of the participants was 71.83 years (SD = 5.90). Among the participants, 58.6% were female, 53.4% were of Han ethnicity, 39.3% were illiterate, and 42.4% reported a monthly per capita income of RMB 1,001–3,000. Most participants were farmers in terms of previous occupation 73.2%. Most participants reported never smoking 80.7%, whereas 76.5% reported alcohol consumption, and 27.8% reported no regular physical exercise. In terms of health-related characteristics, 44.0% of the participants reported fair sleep quality, 32.5% had three or more chronic diseases, and 38.4% had a disease duration of more than 10 years. Detailed sociodemographic and disease-related characteristics are shown in Table 1.

**Table 1:** Comparison of EQ-5D utility scores among rural empty-nest older adults with chronic diseases by sociodemographic and other characteristics (*n* = 425).

Variables	*N*(%)	EQ-5D value P50(P_2_5, P75)	z*/H*	*P*
Age (years), mean (SD)	71.83 (5.90)			
Gender			−0.173	0.863
Male	176 (41.4%)	0.734 (0.363,0.942)		
Woman	249 (58.6%)	0.746 (0.363,0.934)		
Age			29.213	**< 0.001**
60~69	185 (43.5%)	0.827 (0.457,0.942)		
70~79	169 (39.8%)	0.782 (0.363,0.938)		
≥80	71 (16.7%)	0.562 (0.309,0.734)		
Ethnicity			−3.185	**0.001**
Han	227 (53.4%)	0.824 (0.363,0.942)		
Hui	198 (46.6%)	0.701 (0.363,0.876)		
BMI			12.008	**0.007**
Underweight	44 (10.4%)	0.459 (0.269,0.774)		
Normal	247 (58.1%)	0.751 (0.363,0.942)		
Overweight	101 (23.8%)	0.783 (0.421,0.942)		
Obesity	33 (7.8%)	0.655 (0.451,0.875)		
Education level			11.582	**0.003**
Illiterate	167 (39.3%)	0.714 (0.363,0.893)		
Primary school	138 (32.5%)	0.727 (0.363,0.893)		
Junior high school and above	120 (28.2%)	0.827 (0.528,0.942)		
Previous occupation			5.387	0.146
Government or public institution employee	33 (7.8%)	0.824 (0.465,0.975)		
Farmer	311 (73.2%)	0.733 (0.363,0.893)		
Self-employed worker	48 (11.3%)	0.805 (0.363,0.985)		
Freelancer or other occupations	33 (7.8%)	0.734 (0.363,0.877)		
Marital status			−2.433	**0.015**
Unmarried/divorced/widowed	143 (33.6%)	0.659 (0.363,0.893)		
Married	282 (66.4%)	0.756 (0.432, 0.942)		
Family monthly income per capita (RMB)			19.779	**< 0.001**
≤ 1 000	167 (39.3%)	0.691 (0.363,0.893)		
1 001~3 000	180 (42.4%)	0.740 (0.379,0.893)		
3 001~5 000	61 (14.4%)	0.886 (0.616,1.000)		
>5 000	17 (4.0%)	0.934 (0.371,1.000)		
Medical insurance			7.486	**0.024**
None	47 (11.1%)	0.511 (0.216,0.827)		
Employee insurance	72 (16.9%)	0.802 (0.367,0.940)		
Resident insurance	306 (72%)	0.746 (0.431,0.942)		
Exercise frequency			8.112	**0.017**
Never	118 (27.8%)	0.737 (0.363,0.942)		
1~2 times per week	217 (51.5%)	0.703 (0.363,0.893)		
Almost daily	90 (21.2%)	0.825 (0.582,0.942)		
Smoking			−0.115	0.909
Yes	82 (19.3%)	0.734 (0.363,0.942)		
No	343 (80.7%)	0.740 (0.370,0.934)		
Drinking			−0.824	0.410
Yes	325 (76.5%)	0.783 (0.363,0.942)		
No	100 (23.5%)	0.734 (0.363,0.934)		
Self-rated sleep quality			31.137	**< 0.001**
Poor	74 (17.4%)	0.540 (0.340, 0.769)		
Fair	187 (44.0%)	0.734 (0.363, 0.893)		
Good	164 (38.6%)	0.862 (0.565, 1.000)		
Number of chronic diseases			24.540	**< 0.001**
1	190 (44.7%)	0.862 (0.513,0.942)		
2	97 (22.8%)	0.746 (0.394,0.893)		
≥3	138 (32.5%)	0.623 (0.363,0.825)		
Duration of illness (years)			21.327	**< 0.001**
< 5 years	157 (36.9%)	0.862 (0.553,0.942)		
5~10 years	105 (24.7%)	0.734(0.561,0.885)		
>10 years	163 (38.4%)	0.585 (0.363,0.886)		

Bold values indicate statistically significant differences (*P* < 0.05).

### 3.2 Health utility and associated factors among rural empty-nest older adults with chronic diseases

The results showed that health-related quality of life (health utility) scores differed significantly across groups defined by age, ethnicity, BMI, educational level, marital status, family monthly income per capita, medical insurance, exercise frequency, self-rated sleep quality, number of chronic diseases, and disease duration (*P* < 0.05). Details are shown in Table 1.

### 3.3 Correlations among social capital, health-related quality of life, and self-efficacy

**Figure 1:**
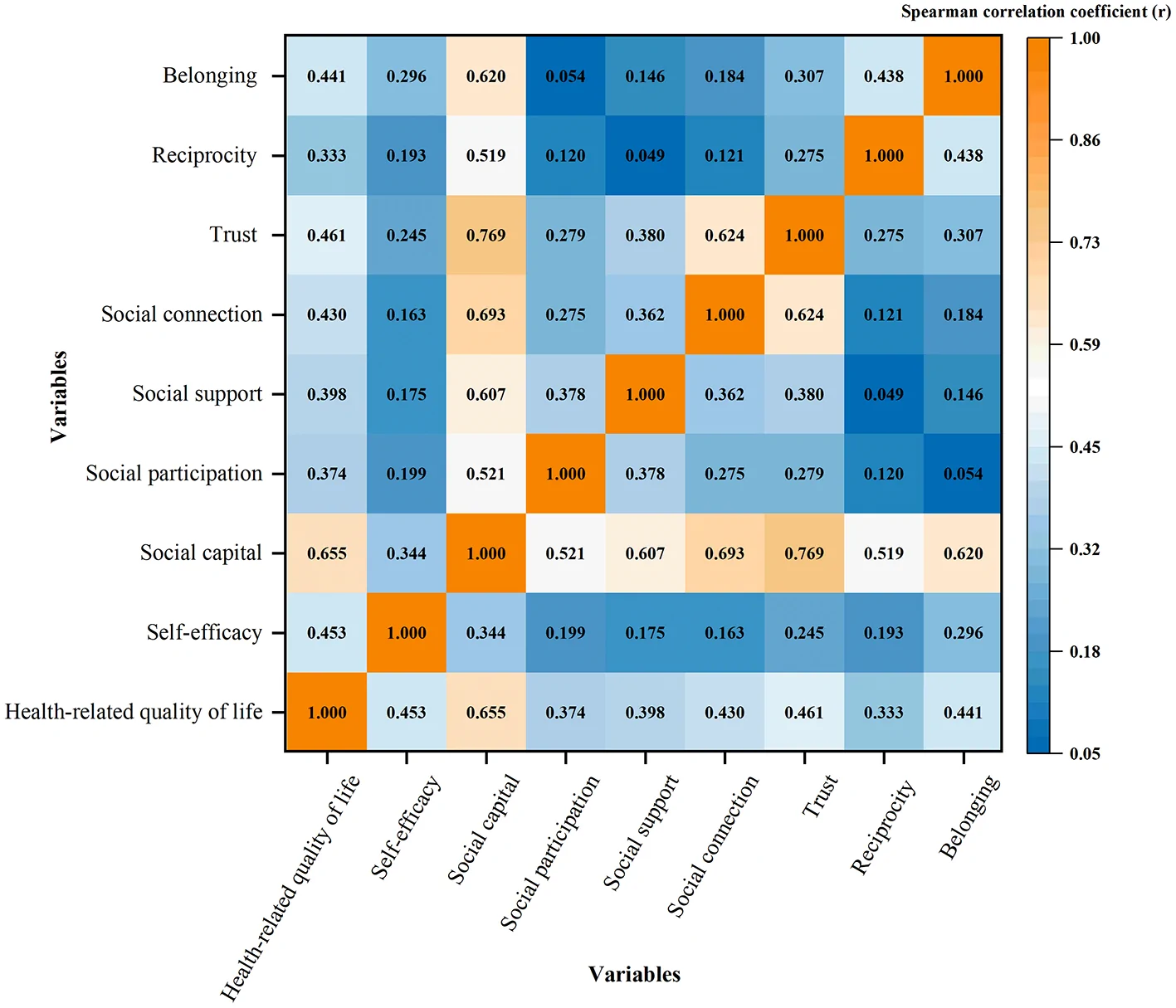
Spearman correlation heatmap among social capital, self-efficacy, and health-related quality of life.

Harman's single-factor assessment revealed seven distinct factors featuring eigenvalues exceeding unity; the primary factor contributed just 33.42% of the total variance, falling short of the 40% threshold, indicating that common method bias failed to exert a substantial influence on the findings. The normality test indicated that the HRQoL scores did not follow a normal distribution (*P* < 0.05). After controlling for the covariates identified as significant in the univariate analyses, partial correlation analyses showed that social capital was positively correlated with HRQoL (*r* = 0.655, *P* < 0.001) and self-efficacy (*r* = 0.344, *P* < 0.001), and HRQoL was positively correlated with self-efficacy (*r* = 0.453, *P* < 0.001). The results are presented in Figure 1.

### 3.4 Latent profile analysis of social capital

**Figure 2:**
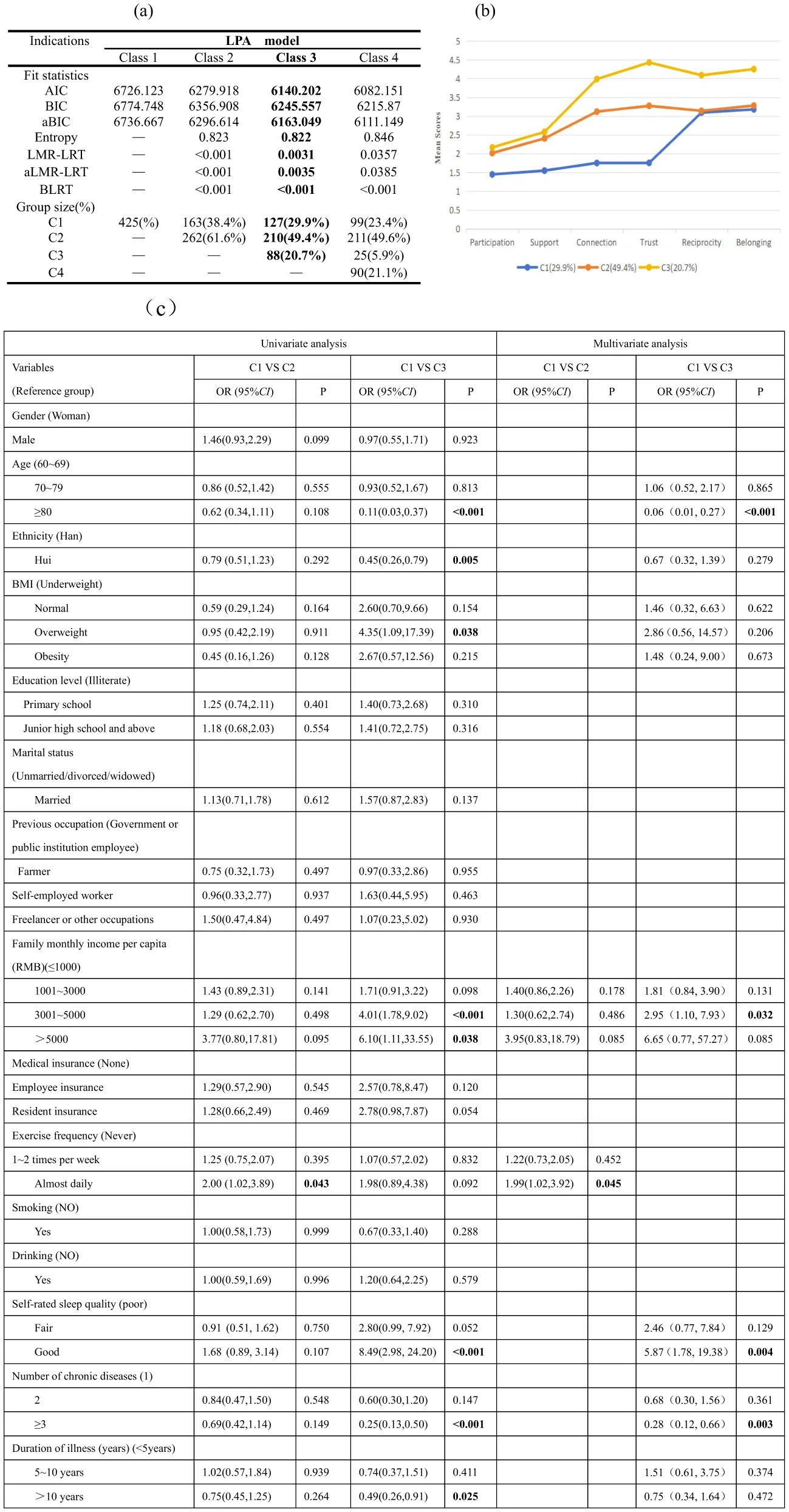
**(a)** Fitting statistics and group size of latent profile analysis. **(b)** Patterns for three distinct profiles. **(c**) Univariate and multivariate analyses based on three distinct profiles.

Figure 2a shows the results of the model fitting index. The values of AIC, BIC and aBIC decrease as the number of potential profile categories increases. In addition, the LMR-LRT and BLRT were statistically significant for the two-through four-class solutions (*P* < 0.05), indicating that each successive model provided a better fit than the preceding model. Entropy values for all models exceeded 0.80, suggesting good classification accuracy. However, in the four-class model, one class accounted for only 5.9% of the sample. For model stability, we chose the three-class solution. The three social capital profiles identified by LPA appear in Figure 2b. According to the score patterns specific to each dimension across classes, Class 1 was labeled the “low social capital group” (*n* = 127, 29.9%), Class 2 the “moderate social capital group” (n = 210, 49.4%), and Class 3 the “high social capital group” (*n* = 88, 20.7%). Figure 2c depicts factors associated with different social capital classes. Univariate and multivariable analyses indicated that exercise frequency distinguished between Classes 1 and 2, whereas age, monthly per-capita household income, self-rated sleep quality, and type of chronic disease distinguished between Classes 1 and 3.

### 3.5 Differences in HRQoL and self-efficacy scores across LPA-derived social capital profiles

Between-group comparisons showed that the three LPA-derived social capital classes differed significantly in health-related quality of life and self-efficacy scores (*P* < 0.001). Further pairwise comparisons indicated a graded increase in both HRQoL and self-efficacy from Classes 1 to 3 (*P* < 0.001). Further details are available in Table 2.

**Table 2:** Differences in health-related quality of life and self-efficacy across different classes (*n* = 425).

Variables	Class1(*n* = 127)	Class2(*n* = 210)	Class3(*n* = 88)	*H*	*P*	Mann-Whitney U
	M (P_2_5, P75)	M (P_2_5, P75)	M (P_2_5, P75)			
Health-related quality of life	0.36 (0.15,0.66)	0.75 (0.48, 0.93)	0.94 (0.88, 1.00)	151.731	< 0.001^**^	C1 < C2 < C3
Self-efficacy	2.10 (1.90, 2.60)	2.50 (2.00, 2.90)	2.80 (2.50, 3.15)	52.136	< 0.001^**^	C1 < C2 < C3

M: median; P_2_5, P75: interquartile range. All *post-hoc* pairwise comparisons were conducted using the Mann-Whitney U test with Bonferroni correction, and all comparisons remained statistically significant after correction (^**^p < 0.001).

### 3.6 Mediation analysis of self-efficacy based on LPA

Considering sociodemographic and disease-related characteristics associated with HRQoL scores, as well as determinants of distinct social capital classes, we included age, ethnicity, BMI, educational level, marital status, monthly household income per capita, medical insurance type, exercise frequency, self-rated sleep quality, number of chronic diseases, and disease duration (years) as covariates. Social capital was categorized through virtual coding, with the “low social capital class (Class 1)” serving as the reference group. A multiple mediation model was constructed with self-efficacy as the mediating variable and HRQoL as the dependent variable. The analysis produced these results (95% bootstrap confidence intervals): (1) moderate social capital group, direct effect (0.002, 0.112), indirect effect (−0.002, 0.053), and total effect (0.020, 0.143), suggesting that the mediating role of self-efficacy between moderate social capital and HRQoL was not supported; and (2) high social capital group, direct effect (0.149, 0.287), indirect effect (0.028, 0.095), and total effect (0.203, 0.353), indicating that self-efficacy mediated the association between high social capital and HRQoL. Further information is outlined in Table 3.

**Table 3:** The mediating effect of self-efficacy on health-related quality of life.

Variables	*B*	*SE*	*T*	*P*	*LLCI*	*ULCI*	*R^2^*
Outcome variable: self-efficacy(low social capital as reference)							0.183
Moderate social capital	0.101	0.057	1.753	0.080	–0.012	0.214	
High social capital	0.269	0.073	3.672	< 0.001	0.125	0.413	
Age	–0.138	0.041	–3.416	0.001	–0.218	–0.059	
Ethnicity	–0.058	0.062	–0.934	0.351	–0.179	0.064	
BMI	0.018	0.038	0.462	0.644	–0.057	0.093	
Education level	0.058	0.040	1.446	0.149	–0.021	0.136	
Marital status	0.015	0.062	0.236	0.813	–0.107	0.136	
Family monthly income percapita	0.039	0.039	1.008	0.314	–0.037	0.115	
Medical insurance	–0.049	0.057	–0.864	0.338	–0.160	0.062	
Exercise frequency	0.074	0.041	1.811	0.071	–0.006	0.154	
Self-rated sleep quality	0.130	0.032	4.077	< 0.001	0.068	0.193	
Number of chronic diseases	0.003	0.035	0.072	0.943	–0.066	0.071	
Duration of illness	–0.050	0.033	–1.503	0.134	–0.114	0.015	
Outcome variable: health-related quality of life(low social capital as reference)							0.395
Moderate social capital	0.057	0.028	2.045	0.042	0.002	0.112	
High social capital	0.218	0.035	6.211	< 0.001	0.149	0.287	
self-efficacy	0.221	0.023	9.504	< 0.001	0.175	0.267	
Age	–0.026	0.019	–1.363	0.174	–0.065	0.012	
Ethnicity	0.012	0.029	0.418	0.676	–0.045	0.069	
BMI	0.009	0.018	0.491	0.623	–0.027	0.044	
Education level	0.009	0.019	0.462	0.645	–0.028	0.046	
Marital status	0.011	0.029	0.378	0.705	–0.046	0.068	
Family monthly income percapita	0.005	0.018	0.294	0.769	–0.030	0.041	
Medical insurance	–0.001	0.027	–0.052	0.958	–0.054	0.051	
Exercise frequency	0.017	0.019	0.864	0.388	–0.021	0.055	
Self-rated sleep quality	0.025	0.015	1.633	0.103	–0.055	0.055	
Number of chronic diseases	–0.022	0.017	–1.308	0.192	–0.054	0.011	
Duration of illness	–0.054	0.016	–3.447	0.001	–0.084	–0.023	
*Effect type*	*Variables*	*Effect*	$s_{\bar{x}}$		*LLCI*	*ULCI*	
Direct and indirect effect of social capital on health-related quality of life (low social capital as reference)							
Direct effect	Moderate social capital	0.057	0.028		0.002	0.112	
	High social capital	0.218	0.035		0.149	0.287	
Indirect effect	Moderate social capital	0.024	0.014		−0.002	0.053	
	High social capital	0.059	0.017		0.028	0.095	
Total effect	Moderate social capital	0.082	0.031		0.020	0.143	
	High social capital	0.278	0.038		0.203	0.353	

SE, standard error; LLCI, lower level of confidence interval; ULCI, upper level of confidence interval.

## 4 Discussion

This study highlights the diversity of social capital. The research results indicate that participants in the high social capital group significantly outperformed those in the low social capital group in terms of HRQoL and self-efficacy. Mediation analysis further demonstrated that self-efficacy mediated the association between high social capital and HRQoL, whereas this indirect effect was not observed in the moderate social capital group. These findings suggest that the relationship between social capital and health-related quality of life may differ across subgroups and may help inform targeted interventions for rural empty-nest older adults with chronic diseases.

Latent profile analysis identified three distinct social capital classes among rural empty-nest older adults with chronic diseases: Class 1 (low social capital, 29.9%), Class 2 (moderate social capital, 49.4%), and Class 3 (high social capital, 20.7%), which supported our first hypothesis. The distribution of classes suggested that, in rural Ningxia, where regional context and social structure may substantially shape social resources, approximately one-third of participants were characterized by low social capital, nearly half by moderate social capital, and only about one-fifth by high social capital. This pattern was broadly consistent with previous findings (29). Moreover, social capital characteristics differed across the three classes.

Class 1 (29.9%) was characterized by low social capital, with consistently low scores across all dimensions, particularly in social participation, social support, social connection, and trust. This class may represent a priority group for social capital–oriented interventions. Class 2 (49.4%) represented a moderate social capital profile, with relatively higher levels of trust and sense of belonging, but persistent deficits in participation and support. This class may represent a subgroup with substantial potential for improvement. Class 3 (20.7%) reflected high social capital, with consistently high scores across all dimensions and notably higher levels of social connection and trust than the other two classes. These profile differences may be associated with geographic context and service accessibility, which may be related to opportunities for participation and social connection; the availability and continuity of family and community support, which may be related to perceived support and sense of belonging; and individual health status, which constrains activity range and social interaction. In particular, individuals in Class 1 may be more likely to experience compounded disadvantages, including remote residence, limited family support, and poorer health, whereas those in Class 3 may benefit from greater residential convenience, stronger family support, and better functional capacity. Together, these factors may be related to differences in social capital accumulation. Prior evidence demonstrates that insufficient social capital is linked to poorer psychological health, reduced capacity to cope with illness, and lower quality of life among rural empty-nest older adults, and may also limit their willingness and ability to seek and utilize health and social support services (30).

Accordingly, social capital interventions in rural Ningxia should be tailored to local geographic and sociocultural conditions. Potential strategies include establishing neighborhood mutual-aid groups, promoting a grid-based community management model integrated with mutual-support care services for older adults, and improving service reach through mobile cultural services or remote participation platforms. In parallel, age-friendly public space planning within rural development initiatives may help strengthen social connectedness, participation, and sense of belonging, thereby enhancing social capital and supporting healthy aging and aging in place.

Furthermore, the heterogeneity analysis shed light on how sociodemographic and disease-related factors play into social capital class categorizations, building upon existing research in this area. Regarding exercise frequency, participants who reported exercising almost daily were more likely to belong to the moderate social capital class, consistent with Gao et al. (31). A plausible explanation is that regular daily exercise, such as morning group exercise or square dancing, may increase opportunities for social participation and foster trust within peer networks. However, limited family support and a relatively narrow social circle related to the empty-nest context may constrain gains in social support and network integration, thereby limiting progression to a higher social capital profile. Participants aged ≥80 years were more likely to be classified into the low social capital class, in line with Pan et al. (32), suggesting an age-related decline in social capital. Increasing age is often accompanied by functional limitations and contraction of social networks, which can reduce participation and weaken trust-based ties. Inadequate support may further contribute to avoidance and social withdrawal, indicating the need for targeted attention and assistance. Higher monthly per-capita household income was associated with membership in the high social capital class, likely reflecting greater financial capacity and resource access that facilitate sustained engagement in community interactions, the establishment and maintenance of trust-based relationships, and adherence to reciprocity norms, thereby supporting the accumulation and consolidation of social capital (33). Participants reporting good self-rated sleep quality were more likely to be classified into the high social capital group. This finding aligns with previous evidence showing that lower social capital is significantly associated with insufficient sleep duration (34). Higher social capital may contribute to better sleep by providing stronger social connections and greater opportunities for social participation, thereby alleviating loneliness and psychological stress. In addition, older adults with three or more chronic conditions were more likely to belong to the low social capital group, consistent with prior studies (35). Multimorbidity is typically associated with poorer health status and reduced capacity for social engagement, which may substantially limit the ability to acquire and sustain social capital.

In summary, the discriminatory effects of factors such as age, exercise, income, sleep, and number of chronic diseases on social capital classes in rural empty-nest older adults with chronic diseases exhibit high directional consistency with previous findings in non-rural or general older adult populations. This indicates that the influence of these sociodemographic and health factors on social capital has a certain degree of generalizability. The present study extends this line of research to the rural empty-nest context, which is characterized by relatively scarce resources and weak family support, thereby providing evidence from a different ecological background for this field. Accordingly, local governments may leverage village committees, village clinics, and public activity venues to organize regular group-based health activities and strengthen neighborhood mutual-aid initiatives, thereby reinforcing informal support networks. Healthcare providers should prioritize home visits for the very old adults and multimorbid empty-nest older adults, encourage participation in appropriate social activities, and facilitate linkage to local healthcare resources to support the accumulation and maintenance of social capital, with the ultimate goal of improving HRQoL.

The present study found that self-efficacy mediated the association between high social capital and HRQoL among rural empty-nest older adults with chronic diseases, whereas the corresponding indirect effect was not observed in the moderate social capital group, partially supporting Hypothesis 3. In this study, self-efficacy was positively associated with both social capital and HRQoL. In the high social capital group, greater social support, neighborhood reciprocity, and better access to resources may provide a more supportive environment for disease coping and positive reinforcement, which may help strengthen self-efficacy. Higher self-efficacy, in turn, may increase confidence in managing daily activities and promote engagement in self-care behaviors, contributing to better HRQoL (36). This pattern is consistent with Social Cognitive Theory, which posits that supportive environments enhance perceived capability and facilitate adaptive health behaviors, ultimately improving quality of life (37). In contrast, the lack of a significant mediation effect in the moderate social capital group may be explained by the relatively uneven composition of social capital in this subgroup, although trust and sense of belonging were relatively higher, social participation and support remained insufficient in this rural empty-nest population, given limited family support, dispersed settlements, and restricted opportunities for community participation. Under such conditions, social capital may not have been sufficiently linked to efficacy beliefs relevant to chronic disease management. However, this interpretation remains hypothetical, as the present study did not directly test the mechanisms related to social participation, social support, or a potential threshold effect of social capital. This interpretation is broadly consistent with evidence suggesting that belonging and trust alone may not be sufficient to support better quality of life when participation and practical support are limited (38). Moreover, rural older adults in general tend to have lower social capital, poorer economic conditions, and insufficient disease-related knowledge (39). Meanwhile, rural areas also lag behind in terms of healthcare resources, social security, and living environment (40). This situation reflects chronic underinvestment by the government in rural public services and precisely highlights the importance of studying the rural empty-nest older adult population. All these factors may undermine older adults' confidence in disease management and may partly account for the absence of the observed indirect association through self-efficacy in the moderate social capital group.

Given that the high social capital group has obvious advantages in social networks, trust, and resource integration, this group may have a relatively unobstructed self-efficacy-related pathway and may therefore serve as a “leveraging point” for intervention. For the moderate and low social capital groups, relying solely on individual self-efficacy enhancement is unlikely to be effective, and priority should be given to repairing their disrupted social support chains. Considering the relatively limited government investment in rural infrastructure and community health services, in practice, healthcare workers should identify patients with different social capital levels in a targeted manner and implement tiered, class-based interventions. For the high social capital group, psychological empowerment interventions focusing on self-efficacy can be strengthened, such as peer modeling and success-experience sharing in chronic disease management that leverage their existing social networks (fully utilizing the “peer modeling spill-over effect” of the high-capital group), thereby amplifying the mediating role of self-efficacy and continuously improving their health-related quality of life. For the moderate and low social capital groups, priority should be given to expanding their social support resources. Here, peer interventions can effectively fill the gap left by insufficient government investment: by organizing village-based health mutual aid groups (peer support), guiding participation in collective health education activities, and building neighborhood social networking platforms (peer support networks), social capital levels can be raised first, and then self-efficacy promotion strategies can be gradually integrated, forming a virtuous cycle of “social capital enhancement—self-efficacy increase—quality of life improvement”, ultimately achieving better health-related quality of life. In other words, in the resource-poor rural empty-nest context, peer intervention is a low-cost, highly feasible strategy that can partially replace or supplement the lack of to-down public services.

## 5 Conclusion

In conclusion, rural empty-nest older adults with chronic diseases exhibit heterogeneous profiles of social capital, which are associated with differences in HRQoL. The relationship between high social capital and HRQoL is mediated by self-efficacy, whereas no such indirect effect is found in the moderate social capital group. These findings suggest that interventions should be tailored to different social capital profiles, with attention to both social resource enhancement and self-efficacy promotion. In resource-limited rural settings, peer-based and community-supported interventions may represent feasible approaches to improving social capital, self-efficacy, and ultimately HRQoL among empty-nest older adults with chronic diseases.

## 6 Strengths and limitations

To our knowledge, few studies have examined social capital heterogeneity among rural empty-nest older adults with chronic diseases, and evidence on how different social capital profiles affect HRQoL through self-efficacy remains limited. A key strength of this study is the combined use of latent profile analysis and mediation analysis, which identified distinct social capital profiles and showed that self-efficacy mediated the association between high social capital and HRQoL, whereas this indirect effect was not observed in the moderate social capital group. These findings provide new evidence for understanding social capital heterogeneity and its psychological pathway to HRQoL in this vulnerable population, and suggest the need for profile-specific interventions. In resource-limited rural empty-nest settings, peer-based and community-supported strategies may be feasible approaches to enhancing social capital, strengthening self-efficacy, and improving HRQoL. Future studies should further develop and test profile-specific interventions that integrate peer support, community participation, and self-efficacy enhancement strategies. However, this study has several limitations. First, this was a cross-sectional study, which can only identify associations among the research variables but cannot infer causal relationships. Future longitudinal studies are needed to track dynamic changes in social capital, self-efficacy, and HRQoL among rural older adults with chronic diseases, and to further clarify the causal pathways among these variables. Second, although a multistage stratified random sampling framework was used, village-level recruitment partly relied on convenience sampling, which may have introduced selection bias and limited the representativeness and generalizability of the findings. Future studies should adopt more rigorous probability sampling methods and expand recruitment to broader rural populations to improve representativeness. Third, due to limitations in the study design and research resources, this study mainly focused on sociodemographic and disease-related variables, while other potential confounding factors, such as participants' mental health status, levels of anxiety and depression, disability status, and objective indicators of chronic disease severity, were not included. In addition, sleep quality was assessed using a brief subjective evaluation rather than a standardized sleep quality scale, which may have introduced measurement bias and limited the accuracy and comparability of sleep quality classification. These factors may have influenced the findings. Future studies should incorporate more precise mental health assessment tools, disability-related indicators, multidimensional disease assessment methods, and standardized sleep quality scales to provide a more comprehensive evaluation of these associations.

## Data Availability

The raw data supporting the conclusions of this article will be made available by the authors, without undue reservation.
